# *Rhizomucor* and *Scedosporium* Infection Post Hematopoietic Stem-Cell Transplant

**DOI:** 10.1155/2011/830769

**Published:** 2011-04-05

**Authors:** Dânia Sofia Marques, Carlos Pinho Vaz, Rosa Branca, Fernando Campilho, Catarina Lamelas, Luis Pedro Afonso, Manuel Jacome, Eduardo Breda, Eurico Monteiro, António Campos Júnior

**Affiliations:** ^1^Bone Marrow Transplantation Service, Instituto Português de Oncologia Francisco Gentil, Rua Dr. António Bernardino de Almeida, 4200-072 Porto, Portugal; ^2^Microbiology Service, Instituto Português de Oncologia Francisco Gentil, Rua Dr. António Bernardino de Almeida, 4200-072 Porto, Portugal; ^3^Pathology Service, Instituto Português de Oncologia Francisco Gentil, Rua Dr. António Bernardino de Almeida, 4200-072 Porto, Portugal; ^4^Otorhinolaringology Service, Instituto Português de Oncologia Francisco Gentil, Rua Dr. António Bernardino de Almeida, 4200-072 Porto, Portugal

## Abstract

Hematopoietic stem-cell transplant recipients are at increased risk of developing invasive fungal infections. This is a major cause of morbidity and mortality. We report a case of a 17-year-old male patient diagnosed with severe idiopathic acquired aplastic anemia who developed fungal pneumonitis due to *Rhizomucor sp.* and rhinoencephalitis due to *Scedosporium apiospermum* 6 and 8 months after undergoing allogeneic hematopoietic stem-cell transplant from an HLA-matched unrelated donor. Discussion highlights risk factors for invasive fungal infections (i.e., mucormycosis and scedosporiosis), its clinical features, and the factors that must be taken into account to successfully treat them (early diagnosis, correction of predisposing factors, aggressive surgical debridement, and antifungal and adjunctive therapies).

## 1. Introduction

Hematopoietic stem-cell transplant (HSCT) recipients are at increased risk of developing invasive fungal infections (IFIs). This is a major cause of morbidity and mortality. Invasive aspergillosis (IA) remains the most commonly reported IFIs among these patients, accounting for nearly 60% of all the IFIs, with a mortality rate of 60–85% (mostly in the postengraftment period after HSCT) [1–3]. Nowadays, we are noticing a change among the profile of the filamentous IFIs, with an increase in the incidence of IFIs by moulds other than *Aspergillus* species. They, now, account for nearly 14% of all IFIs (half of these are caused by *Mucorales spp.*, followed by *Fusarium spp.*) [3, 4]. Perhaps the widespread usage of new antifungal therapies might contribute for the emergence of these unusual IFIs [4]. These infections are very difficult to diagnose in an early stage, which makes treatment a challenge and overall mortality rate very high. We report a case of a 17-year-old male patient who developed fungal pneumonitis due to *Rhizomucor sp.* and rhinoencephalitis due to *Scedosporium apiospermum* 6 and 8 months after undergoing allogeneic HSCT from an HLA-matched unrelated donor.

## 2. Case Report

**Figure 1:**
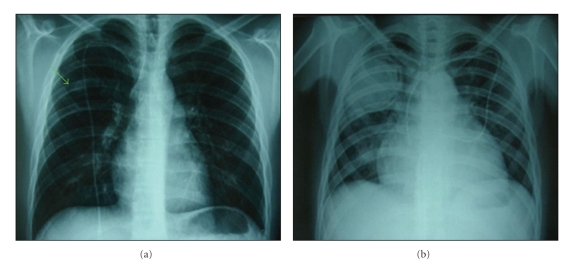
Thoracic X-ray: (a) on day 176, it could be observed a small pulmonary node behind the fifth right thoracic rib (arrow); (b) 3 months, later the pulmonary node was much larger and became cavitated.

**Figure 2:**
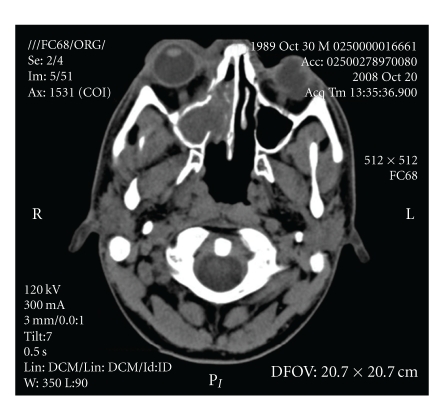
CT scan of the perinasal sinuses: infiltrative lesion of the perinasal sinuses that exerts compression on the right optic nerve.

**Figure 3:**
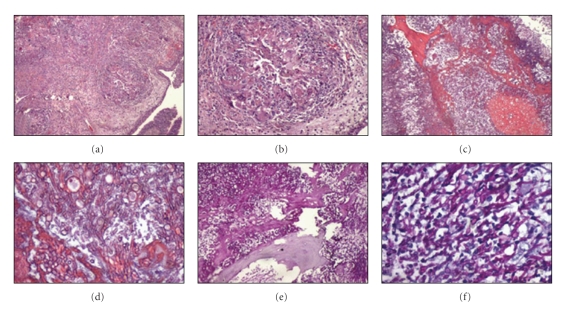
Ethmoiditis due to* Scedosporium apiospermum*. (a) Mucosa lined by respiratory type epithelium with intense inflammatory reaction (hematoxylin and eosin [H&E] staining, magnification ×100); (b) higher magnification (×400) of (a) it can be identified numerous epithelioid granulomas and multinucleated giant cells; (c) partially destroyed bone tissue and numerous fungal hyphae (H&E staining, magnification ×100); (d) morphologically the fungal hyphae resemble *Aspergillus spp*. (H&E, magnification ×400); (e) bone tissue destruction by fungal hyphae (PAS staining, magnification ×100); (f) the main host response is a mixed neutrophilic and monocytic infiltrate (PAS staining, magnification ×400).

A 17-year-old Caucasian male was diagnosed with severe idiopathic acquired aplastic anemia in January 2007. He had no related genotypically matched donor for HSCT, so he underwent a 5-month course of therapy with cyclosporine and antithymocyte globulin with no response. In February 2008, he received an allogeneic HSCT from an HLA-matched unrelated donor (10/10 HLA antigens). The preparative regimen consisted of alemtuzumab, fludarabine, and cyclophosphamide. Graft-versus-host disease (GVHD) prophylaxis consisted of tacrolimus and methotrexate (starting day 1). He showed evidence of hematopoietic engraftment on day 17. Digestive tract GVHD (biopsy proven) developed on day 25 and was treated with corticosteroids (prednisone at 1 mg/Kg twice a day) and tacrolimus, with gradual resolution. The evaluation on day 33 showed complete chimerism and a normal bone marrow. He was discharged on day 41 with tacrolimus, oral prednisone and antifungal prophylaxis with fluconazole 400 mg/day. His outpatient course was complicated by gradual development of leucopenia, requiring granulocyte colony-stimulating-factor therapy. On day 115, the patient was admitted to the hospital for febrile neutropenia with cough and odynophagia. He received antibiotic therapy with piperacillin plus tazobactam and amikacin although no infectious agent was isolated. Cytomegalovirus and Epstein-Barr virus infections were also excluded. The day 119 evaluation showed complete chimerism and normal bone marrow. He recovered and was discharged on day 125. He did well until day 165, when he was admitted for an acute tonsillitis with febrile neutropenia. A cervical computerized tomography (CT) showed an abscess in the peritonsillar space. A tonsillar biopsy was made which revealed a polymorphic infiltrate eosinophil-rich and no other findings. No infectious agent was isolated. He received antibiotic therapy with piperacillin plus tazobactam and antifungal prophylaxis with posaconazole. As he maintained fever, clindamycin was added to therapy in order to increase the synergistic effect against anaerobes (essentially mouth anaerobes that are partially covered by piperacillin and tazobactam) and to increase peritonsillar tissue antibiotic diffusion, thus allowing methicillin-resistant *Staphylococcus aureus* coverage. There was resolution of the febrile episode and the patient was discharged on day 179. At this time, he maintained GVHD therapy with steroids and tacrolimus and antifungal prophylaxis with posaconazole (200 mg three times daily). Close followup was done. Although somewhat better, the patient maintained persistent complaints of cough and serous sputum. The haematological values were stable. On the thoracic X-ray (Figure 1(a)) there was small pulmonary node that gradually enlarged so he forwarded a thoracic CT, on day 192 which revealed a cavitated lesion on the right superior pulmonary lobe. Galactomannan antigen was negative. Bronchoscopy and bronchoalveolar lavage (BAL) were performed. Thoracic surgery was proposed, but the patient was considered not fit to such intervention. At this time, antifungal treatment with voriconazole was started. Nevertheless, the patient's clinical condition began to deteriorate with the development of persistent cough (no differences on sputum), dyspnea, headaches, otalgia, fever, and neutropenia. On day 211 he was admitted to the hospital. The patient developed hemoptysis and acute respiratory and renal failure, so he required intensive care unit (ICU) admission. The fungal culture result of the BAL revealed a *Rhizomucor sp.* infection on day 215. Liposomal amphotericin B and caspofungin combination therapy was started. The patient started to get better, and he was discharged from the ICU to the ward on day 223. However, he maintained persistent fever and the pulmonary cavitated nodule continued to get worse on thoracic X-ray image (Figure 1(b)). On day 237, he started to complain of right periorbital edema and gradually developed sinusitis, exoftalmia, and amaurosis of the right eye. CT scan of the perinasal sinuses revealed an infiltrative lesion of the perinasal sinuses with ethmoiditis and compression of the right optic nerve (Figure 2). Ethmoidectomy was performed on day 264. Pathology analysis showed signs of ethmoiditis and numerous fungal hyphae (Figures 3(a)–3(f)). Microbiological analysis revealed fungal infection due to *Scedosporium apiospermum*. As his clinical condition continued to deteriorate, antifungal combination therapy was changed to posaconazole along with liposomal amphotericin B, but no response was obtained. On day 310, he started to complain of persistent headache, and on day 322, he developed left hemiparesis and dysarthria probably due to rhinoencephalitis. Consciousness became gradually depressed and death overcame on day 324 post HSCT.

## 3. Discussion

This case highlights the emergence of two unusual filamentous IFIs in a young patient who received an allogeneic HSCT from an unrelated HLA-matched donor, first with a pulmonary mucormycosis and then with a rhinocerebral scedosporiosis. *Rhizomucor sp.* belongs to the order *Mucorales*. Recent reclassification has abolished the order Zygomycetes and placed the order Mucorales in the subphylum Mucormycotina. Therefore, we refer to infection caused by Mucorales as mucormycosis, rather than zygomycosis [5, 6]. The genus *Scedosporium *consists of two medically important species: *Scedosporium apiospermum *(and its teleomorph or sexual state *Pseudallescheria boydii*) and *Scedosporium prolificans*. These mould infections are known as scedosporiosis [7, 8]. Invasive mucormycosis and scedosporiosis are unusual and highly lethal IFIs that can arise in the postengraftment period of HSCT. Isolated, they can achieve a mortality rate superior to 90% in this kind of patients [9, 10]. To our knowledge, there have been few reports of these two IFIs arising in the same patient [11, 12]. These moulds can be widely recovered from the environment [5, 7, 8]. A common route of acquisition is the respiratory tract through inhalation of infectious spores that may establish an initial infection in the sinuses. Other less common routes of acquisition include the intestinal tract following ingestion or by inoculation through breaches in or penetrating injuries to the skin. Angioinvasion is a prominent feature of these IFIs progressing to tissue necrosis and infarction, and this presumably accounts for their ability to cause rapidly invasive infections sometimes with dissemination [5, 7, 8]. From a clinical standpoint, mucormycosis describes infections characterized by one or more of a triad of rhinocerebral, pulmonary, and disseminated disease [5]. Scedosporiosis represents a broad spectrum of clinical diseases, ranging from transient colonization of the respiratory tract to invasive localized disease (mostly involving the bones and joints but also the skin in which case it is known as mycetoma) and at times disseminated disease [7, 8]. The disseminated form of these IFIs is mostly seen among immunocompromised patients, especially in those with hematologic malignancies during periods of prolonged neutropenia [5, 7, 8]. Innate immune defenses comprising phagocytic responses play a critical role in host defense against these filamentous fungi [5, 7, 8]. So, it seems plausible that host defense defects that occur in HSCT recipients as a result of prolonged neutropenia and lymphopenia have a profound impact on the susceptibility and severity of these IFIs [5, 7]. Other factors could also confer high susceptibility to disseminated disease, such as steroidtherapy (maybe because they cause impaired phagocyte function), GVHD (the greater the extent of mucosal injury the greater the risk of IFIs), and serum iron availability (increased iron facilitates fungal growth), but further studies are needed [2, 5, 7, 8, 13]. It seems that antifungal prophylaxis can also have a selection effect for these IFIs as *Rhizomucor sp.* are resistant to voriconazole therapy, and its usage as prophylactic therapy may cause often breakthrough mucormycosis [4, 14]. *Scedosporium spp. *have been known to emerge as pathogens in patients receiving amphotericin B, fluconazole, or itraconazole [7]. In this reported case, the patient had multiple high-risk factors for the development of IFIs, as he received an allogeneic HSCT from an unrelated donor, had neutropenia for a long period, fever (so he received large-spectrum antibiotic therapy for several times), developed gastrointestinal GVHD, and required chronic immunosupression for GVHD control [2, 5, 7, 8, 13, 15, 16]. Although prophylactic fluconazole therapy is highly effective in reducing IFIs-related morbidity and mortality in these patients, it applies only to fluconazole-susceptible *Candida* species infections (*Candida kruseii* and some strains of *Candida glabatra* are not susceptible to fluconazole therapy) [17–19]. Because invasive candidiasis is more frequent in the pre-engraftment period, we started fluconazole at a dosage of 200–400 mg/day until day+75 [17, 19, 20]. As the patient continued to require high-dose corticosteroid therapy (prednisone >1 mg/kg/day) for digestive tract GVHD control, in the postengraftment period, we changed prophylactic antifungal therapy to posaconazole, according to our service protocol, in order to prevent IA. This protocol is based in a clinical trial published in 2007 by Ullmann et al. This clinical trial enrolled 600 patients and compared fluconazole versus posaconazole prophylaxis for IFIs. It showed a clear benefit toward posaconazole prophylaxis in the prevention of IA (OR = 0.31 [0.13–0.75], *P* < .006) without inferior efficacy in the prevention of IFIs (OR = 0.56 [0.30–1.07], *P* < .07) [21]. Nevertheless, the patient developed a breakthrough pulmonary mucormycosis while on posaconazole prophylaxis. Other similar cases have been reported [22]. In 2010, Winston et al. published a prospective study that evaluated the efficacy, safety, breakthrough infections and antimicrobial resistance of long-term posaconazole prophylaxis in 106 consecutive adult allogeneic HSCT recipients [23]. They reported breakthrough IFIs in 7.5% of patients while on posaconazole within 6 months after HSCT. Only 2 of 9 infecting isolates tested were resistant to posaconazole (both Candida glabrata). Mean peak and trough plasma posaconazole concentrations were relatively low in neutropenic patients with oral mucositis and other factors possibly affecting optimal absorption of posaconazole [23]. In this case report, the isolated *Rhizopus *was not identified at the species level, and its sensibility to posaconazole was not tested. However, other factors may also have affected the serum levels of posaconazole. Tacrolimus could be one of such factors, because its concomitant use can reduce the optimal serum levels of posaconazole [24]. We adjusted tacrolimus dosage according to its serum levels. on the other hand, the patient had digestive tract GVHD. This could also be responsible for reduced serum levels of posaconazole [23, 24]. Therefore, when using prophylactic posaconazole, we must be aware of other factors that may affect its absorption/serum levels and implement strategies to improve posaconazole exposure, including the use of higher doses, administration with an acidic beverage, or restriction of proton pump inhibitors [23]. There are five factors that are crucial to successful treatment of these IFIs. The first one is early diagnosis: to recognize patients at increased risk and early signs of infection. This is the most important and troublesome factor, because it is difficult to make an early diagnosis of these IFIs. Initially, clinical features of pulmonary mucormycosis often resemble that of IA in severely immunocompromised patients such as occurred in this case [5, 8]. However, it is the consideration of mucormycosis or scedosporiosis as a diagnosis that may lead to timely confirmation by successful biopsy/culture of the causative organism [5, 7, 8]. Thoracic CT scan is the most sensitive imagiologic tool to detect early pulmonary IFIs, and, therefore, it must be considered the “gold standard”. Some authors perform weekly CT for early detection of IFIs [25]. However, due to economic reasons, this is not the usual procedure in our service. Ordinarily, these patients are monitored with thoracic X-ray performed twice a week. Thoracic CT is performed only on clinical and/or imagiologic suspicion of IFIs. This approach has several limitations, because thoracic X-ray is too insensitive for the diagnosis of IFIs. In early stages of IFIs, thoracic X-ray findings may be scarce, nonspecific, or even undetectable. Therefore, many IFIs may only be diagnosed in later stages by this technique, thus affecting the prognosis of these patients [25]. Diagnosis can be confirmed by biopsy of affected tissues, when accessible, although cultures may prove negative. Genus/species identification is made by culturing the organism and documenting characteristic morphological features [5, 8]. The problem is that fungal culture identification takes long. Even the findings on pathology of *Scedosporium spp. *are very similar to *Aspergillus spp.* and other hyalohyphomycotic species, and there are no serologic or molecular specific diagnostic procedures available to make correct diagnosis faster [8]. So, we started voriconazole empirically though galactomannan antigen was negative. Obviously, there was no response because *Rhizopus sp.* are resistant to this therapy and the IFIs progressed despite later adequate antifungal therapy. Research is needed in order to discover newer and faster specific diagnostic procedures. It is possible that in the near future, some serologic and molecular specific diagnostic procedures may become available (e.g., peptidorhamnomannam for *Pseudallescheria boydii*) [8]. The second consideration is to remove/reduce any reversible predisposing factor such as, for example, reducing the level of immunosuppression [5]. The third consideration is surgical aggressive debridement as early as possible. Surgical excision of the disease has been a component of the standard of care and should be considered whenever possible as it improves survival [5, 7, 8]. This is the second most important message to remember from this case report: surgery cannot be delayed and must be done as early as possible to achieve benefit as long as IFIs are suspected. Even among immunocompetent individuals, infections caused by these agents usually require extensive debridement and sometimes amputation to achieve cure [8]. For these reasons, extensive plastic surgery may be required [5]. The fourth consideration is antifungal therapy. Unfortunately, because the relative rarity of these infections, the choice of antifungal therapy is less well established, and it has been based on experience, supplemented by information gleaned from animal model studies and *in vitro* susceptibility data. Monotherapy with liposomal amphotericin B has been a classical choice for mucormycosis. Doses in the range of 10–15 mg/kg/day have been used although the optimal dose remains unclear [5]. Posaconazole can also be an alternative to this IFIs [26, 27]. Once again, the optimal dosage is unknown. Dosage of 800 mg/day in divided doses has been reported [5]. There are also case reports that describe successful outcomes with combination of liposomal amphotericin B with either caspofungin or posaconazole where single-agent therapy has failed [5, 6, 9]. Caspofungin monotherapy has no *in vitro* activity against Mucorales. Nevertheless, Ibrahim et al. showed, *in vivo*, that caspofungin had significant activity against *Rhizopus oryzae* when it was given prophylactically but not when therapy was started after infection [28]. This study has shown that *Rhizopus oryzae*, the most common pathogen causing mucormycosis, expresses the target enzyme for echinocandins (1,3 beta-glucan synthase) [28]. Though we did not identified the isolated *Rhizopus *at the species level, these studies suggest that caspofungin may have a role in combination therapy against mucormycosis, and, therefore, combination antifungal therapy for mucormycosis should be considered. In the other hand, antifungal therapy with echinocandins seems to be noneffective for *Scedosporium spp.* infections [7, 8]. Newer agents, such as voriconazole, have shown variable results in the treatment of scedosporiosis [8] with a trend toward improved survival when compared to amphotericin B in some studies [7, 8]. *In vitro,* studies have shown a synergistic action from the combination of terbinafine and voriconazole on *S. prolificans* [10]. Overall the susceptibility profile for these two fungal agents is very different and antifungal therapy experience too small. Altogether, they made therapeutic choice a difficult challenge in the setting of this case report. Finally the fifth factor to be considered is adjunctive therapies. These include hyperbaric oxygen (HBO) therapy, iron chelation therapy, and immunotherapy [5, 6, 29, 30]. HBO therapy seemed to improve survival in patients with mucormycosis, provided they receive an adequate course of antifungal therapy in a series of 28 patients. Almost all patients received amphotericin B and major benefit was seen for diabetic patients and for those patients whose predisposing condition was rectified [5, 29]. Deferasirox iron chelation therapy has been reported to show synergy with lipid polyenes against mucormycosis [5, 6, 30]. Although HBO is not worldwide available, deferasirox is a feasible therapy. Interferon-*γ* and granulocyte-macrophage colony-stimulating factor have shown experimentally to increase Mucorales hyphal damage by polymorphs though *Rhizopus sp.* were found to be less susceptible to the host response [5]. This kind of therapies holds promise although experience with them is clearly preliminary and further experimental and clinical studies are needed.

## 4. Conclusion

We are noticing a change among the profile of the filamentous moulds responsible for IFIs in HSCT recipients. Though unusual *Rhizomucor *and *Scedosporium spp.* IFIs are highly lethal and may resemble IA in the clinical field. Prompt diagnosis and early aggressive surgery are key factors to successful treatment of these emergent IFIs because knowledge about their medical treatment is still largely based on animal models, *in vitro* studies, and weak clinical experience. Further research for specific/faster diagnostic procedures, new antifungal drugs, and randomized clinical trials are needed.
